# Microinvasive Fungal Rhinosinusitis: Proposal of a New Subtype in the Classification

**DOI:** 10.3390/jcm9020600

**Published:** 2020-02-24

**Authors:** Min Young Seo, Hyeri Seok, Seung Hoon Lee, Ji Eun Choi, Sang Duk Hong, Seung-Kyu Chung, Kyong Ran Peck, Hyo Yeol Kim

**Affiliations:** 1 Department of Otorhinolaryngology-Head and Neck Surgery, Korea University College of Medicine, Korea University Ansan Hospital, Ansan 15355, Korea; chariseoma@gmail.com (M.Y.S.); shleeent@korea.ac.kr (S.H.L.); 2 Department of Otorhinolaryngology-Head and Neck Surgery, Samsung Medical Center, Sungkyunkwan University School of Medicine, Seoul 06351, Korea; jieun8119.choi@samsung.com (J.E.C.); kkam97@gmail.com (S.D.H.); rhinochung@gmail.com (S.-K.C.); 3 Division of Infectious Diseases, Department of Medicine, Korea University College of Medicine, Korea University Ansan Hospital, Ansan 15355, Korea; hyeri.seok@gmail.com; 4 Division of Infectious Diseases, Department of Medicine, Samsung Medical Center, Sungkyunkwan University School of Medicine, Seoul 06351, Korea; krpeck@skku.edu

**Keywords:** invasive fungal infections, sinusitis, classification, Aspergillus, Rhizopus

## Abstract

Background: Fungal rhinosinusitis (FRS) with mucosal invasion is not classified by the current criteria, and clinical reports on the topic are limited. The aim of this study was to present our 25-year experience on fungal balls with mucosal invasion that do not appear in the FRS classification. Methods: Of 1318 patients who underwent endoscopic surgery with paranasal FRS between November 1994 and July 2019, 372 underwent mucosal biopsies. Medical chart and pathology review were performed on 13 patients diagnosed as having fungal balls with mucosal invasion without accompanying tissue invasion. Results: Histopathologic findings identified all fungi as belonging to the *Aspergillus* species. In 13 patients, 7 fungal balls were located in the maxillary sinus, 3 in the sphenoid sinus, and 3 in both the maxillary and ethmoid sinuses. The median age at diagnosis was 67 years (interquartile range (IQR): 62–72), and the sex ratio was 1:2 (4 men and 9 women). Five patients had comorbidities—three with diabetes mellitus and two with hematologic malignancy—all of whom received postoperative antifungal therapy. The median duration of antifungal treatment was 13 weeks (IQR: 8–17). No recurrences occurred during the median follow-up period of 30 months (IQR: 22–43). Conclusions: Patients who have been clinically diagnosed with a fungal ball and showed mucosal invasion but no vascular invasion, based on pathologic findings after surgery, may need a new FRS classification category, such as microinvasive FRS, and adjuvant antifungal treatment may be needed for immunocompromised patients with microinvasive FRS. Key points: Fungal rhinosinusitis with mucosal invasion is different from fungal ball and invasive fungal rhinosinusitis and may be classified in a separate category as microinvasive FRS.

## 1. Introduction

Fungal rhinosinusitis (FRS) exhibits various clinical characteristics, ranging from colonization to life-threatening invasive disease. FRS classification was first suggested by Hora et al. [1] in 1965, who categorized FRS into invasive and non-invasive groups. The non-invasive group included patients with clinical symptoms similar to those of chronic paranasal rhinosinusitis but who did not respond to routine therapeutic measures, and the invasive group included those classified based on clinical images characterized by a mass involving the surrounding tissues, such as the orbit, nasal cavity, or cheek tissues, with some cases exhibiting overlying skin involvement. The International Society for Human and Animal Mycology classified the invasive and non-invasive types according to histologic findings. According to this classification, invasive diseases include acute invasive (fulminant), granulomatous invasive, and chronic invasive FRS, whereas the non-invasive diseases include saprophytic fungal infestation, fungal balls, and fungus-related eosinophilic FRS, including allergic FRS [2].

Invasive FRS is defined as the presence of vascular invasion and sparse inflammatory reaction associated with the destruction of local structures observed on histopathologic examination. Invasive FRS is divided into subtypes according to the disease duration, whereby acute invasive FRS develops within 4 weeks and chronic invasive FRS lasts more than 12 weeks. Acute invasive FRS is fatal, with an estimated 50% disease-specific mortality, and mainly occurs in immunocompromised hosts [3,4,5,6,7]. A prior study reported neutropenia (absolute neutrophil count < 500/µL) in 82.9% of overall cases of acute invasive FRS [7]. In contrast, chronic invasive FRS is rare, with limited reports on the clinical features and pathophysiology. One study reported that only six cases were finally diagnosed as chronic invasive FRS during 15 years of a multi-institutional study [8]. *Aspergillus* and *Rhizopus* species are the main causative organisms of chronic invasive FRS, and the most common comorbidity was diabetes mellitus [9].

Fungal balls of the paranasal sinus (PNS) are formed through the colonization of fungal hyphae in the PNS without histopathological evidence of parenchymal invasion. Fungal ball is the most common type of FRS found in immunocompetent and immunocompromised hosts, and the main causative organisms are of the *Aspergillus* species [10,11]. In contrast to invasive FRS, fungal balls can be treated by endoscopic sinus surgery (ESS) without the need for additional antifungal treatment, and a previous study reported no recurrence of fungal balls after ESS during follow-up [12].

**Figure 1:**
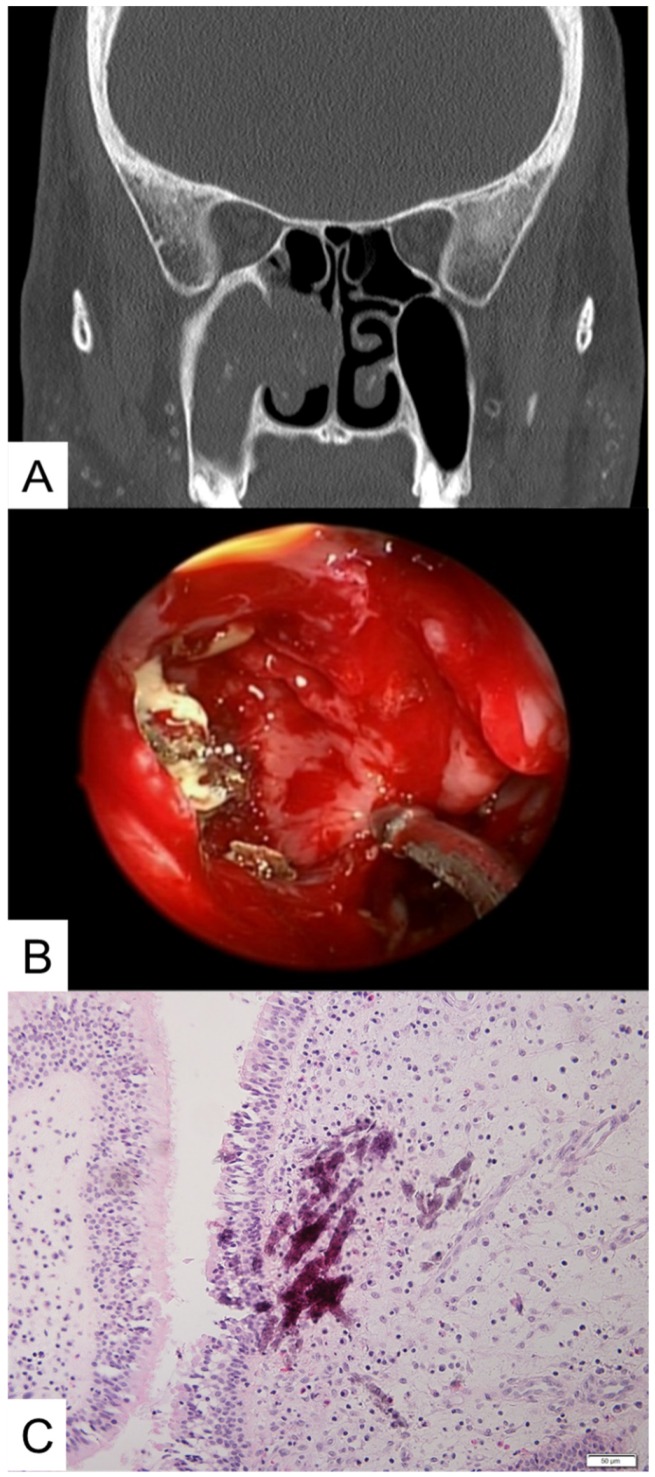
(**A**) Radiologic finding showing a fungal ball located in the right maxillary sinus protruding into the adjacent ethmoid sinus. (**B**) Intraoperative endoscopic evaluation of the right maxillary sinus. Note the fungal ball with thickened mucosa and inflammatory change. (**C**) Pathological confirmation of submucosal invasion of fungal material (hematoxylin and eosin stain, ×200).

Invasiveness is critical in the classification and treatment of FRS. However, we found some cases of fungal balls associated with mild inflammation of the surrounding mucosa confirmed histologically but without direct vascular invasion or involvement of the surrounding structures beyond the submucosa. These cases of FRS were not entirely consistent with the definition of invasive FRS and of fungal balls (Figure 1). Thus, we present 13 cases of fungal balls with only mucosal invasion, encountered during a 25-year period.

## 2. Materials and Methods

This retrospective case–control study was conducted to identify the characteristics of FRS with mucosal invasion. We enrolled patients aged > 18 years who were diagnosed with paranasal FRS using ESS between November 1994 and July 2019 at the Samsung Medical Center, a tertiary-care referral hospital in Seoul, Republic of Korea. These patients were diagnosed with fungal sinusitis based on the review of pathology slides, which confirmed tissue invasion. Complete removal of fungal material using ESS was achieved in all 1318 patients, and additional mucosal biopsies were performed in cases of suspected mucosal inflammation or radiologically proven bony defects (*n* = 372). All mucosal biopsy tissues were rinsed with normal saline before formalin fixation in the operating room to reduce the chances of fungal contamination. All pathology slides were evaluated by a certified pathologist experienced in dealing with sinonasal diseases. All patients with any form of invasion were referred to an infectious disease specialist for consultation regarding additional antifungal treatment. Patients’ demographic data, initial symptoms, medical comorbidities, pathologic reports, and disease-free status and duration were collected at the last follow-up visit. Information on additional treatment after surgery, including antifungal therapy obtained from prescription records, was also collected. To compare the two groups, Pearson χ^2^ tests and Fisher’s exact tests were used for categorical variables and Student’s *t*-test and Mann–Whitney *U* tests were used for continuous variables when appropriate. The study protocol was reviewed and approved by the Institutional Ethics Committee at the Samsung Medical Center (IRB number: SMC 2018-04-119).

## 3. Results

**Figure 2:**
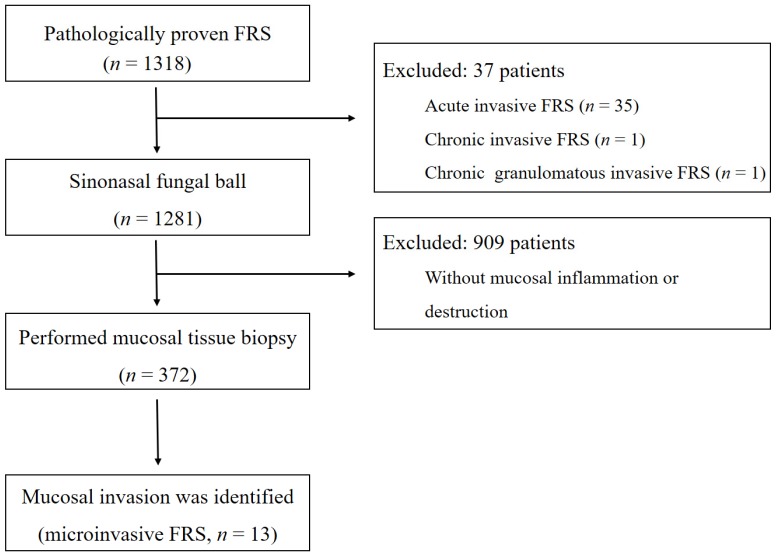
Flowchart representing patient selection.

During the study period, a total of 1318 patients underwent endoscopic sinus surgery for paranasal FRS. Among 1281 patients diagnosed with fungal balls, 374 underwent additional mucosal biopsy. Finally, 13 patients with FRS involving mucosal invasion, not defined by the current classification, were identified (Figure 2). Pathologic findings of these patients included fungal balls with focal invasion to the subepithelial tissue, chronic sinusitis with eosinophils with fungal hyphae in the surface and superficial parenchyma, chronic active sinusitis with fungal hyphae within necrotic and inflammatory exudate and the surface of inflamed granulation tissue, and acute inflammatory exudates and blood clots with fungal hyphae in the mucosa. Clinical characteristics of patients with FRS involving mucosal invasion are shown in Table 1. The median age at diagnosis was 67 years (interquartile range [IQR]: 62–72; 67 to 10), and the sex ratio was 1:2 (4 men and 9 women). Fungal balls were located in the maxillary sinus in seven patients, in the sphenoid sinus in three patients, and in both the maxillary and ethmoid sinuses in three patients. All patients with FRS involving mucosal invasion were identified as having the *Aspergillus* species on pathologic findings. Five patients had no symptoms during their initial visit, whereas eight had nonspecific sinusitis-related symptoms (three with purulent rhinorrhea; two with postnasal drip; and one each with facial pain, headache, and nasal obstruction). The median duration of symptoms was 8 months (IQR: 2–28). In a 1:2 comparison of patients with fungal balls with and without mucosal invasion, there were no significant differences in the demographic and clinical characteristics of patients.

Five patients had comorbidities: three with diabetes mellitus and two with a hematologic malignancy (one acute biphasic leukemia and one diffuse large B-cell lymphoma). The two patients with a hematologic malignancy had no rhinologic symptoms but were diagnosed with fungal balls incidentally during a work-up for bone marrow transplantation. The three patients with diabetes had well-controlled blood glucose levels, made regular endocrinology visits, and had no accompanying diabetic ketoacidosis events. Postoperative antifungal treatment was administered in five patients with a comorbidity as they were considered as immunocompromised hosts. Amphotericin B was used in three patients, voriconazole in one, and itraconazole in one. The median duration of antifungal treatment was 13 weeks (IQR: 8–17). The antifungal agent treatment regimens and durations are shown in Table 1. No recurrences occurred in any patient with fungal balls with mucosal invasion during the median follow-up period of 30 months (IQR: 22–43; 42 to 36).

## 4. Discussion

To the best of our knowledge, this is the first study to evaluate PNS fungal balls with only mucosal invasion, i.e., without angioinvasion. This study is critical in that we share our clinical experience with variant forms of FRS that are not currently classified.

In the diagnosis of FRS, invasiveness is one of the most important factors that determine the treatment modality and disease outcome. Invasive FRS requires immediate antifungal treatment after surgery, whereas in case of fungal balls, endoscopic surgical removal is sufficient without the need for additional antifungal therapy [12]. Fungal balls are defined according to the following criteria: (1) radiologic evidence of sinus opacification with or without calcifications; (2) mucopurulent, cheesy, or clay-like materials within a sinus; (3) dense conglomerated fungal hyphae separate from, but adjacent to, the sinus mucosa; (4) chronic inflammatory response in the mucosa adjacent to fungal elements; and (5) no histologic evidence of fungal invasion of the mucosa, blood vessels, or underlying bone [13]. However, we identified 13 patients with fungal balls who could not be classified according to these current criteria due to adjacent mucosal invasion. No patient showed destruction or invasion of the surrounding bone or soft tissues based on clinical, endoscopic, and radiologic findings; however, mucosal invasion was confirmed in pathologic findings. FRS in these patients was not classified as invasive FRS because of the absence of angioinvasion.

Patients with FRS involving mucosal invasion did not show vascular invasion; however, because of mucosal invasion, we considered that additional antifungal treatment might be necessary. Adjuvant antifungal therapy was used in five patients with comorbidities such as diabetes or hematologic malignancy, and all patients who received antifungal treatment showed no evidence of recurrence during the follow-up period. Because the clinical features of these patients did not differ from those of other patients diagnosed with PNS fungal balls, there was some debate on whether antifungal therapy should indeed be performed in addition to surgery. However, several cases of fungal balls progressing to invasive FRS in immunocompromised patients have been reported [14,15,16]. A prior study reported the case of a patient with sinus fungal ball in the sphenoid, who died 2 weeks after surgery due to carotid invasion; the patient was under long-term steroid therapy for asthma [14]. Thus, in cases of fungal balls without clinically invasive features but with observed mucosal invasion on histopathologic examination, progression to invasive FRS may occur in immunocompromised hosts without additional antifungal treatment.

To the best of our knowledge, there is no concept yet for defining a fungal ball with mucosal invasion; however, we consider that a distinct subtype may be needed for this condition because it has characteristics that differentiate it from invasive FRS. We propose a different subtype called “microinvasive FRS” because preoperative clinical and radiologic findings were similar to those of fungal balls, but, in addition, mucosal invasion was observed in pathologic findings after surgery. Thus, in the presence of mucosal invasion, postoperative antifungal therapy should be considered, especially in immunocompromised patients.

Paknezhad et al. [17] previously reported another intermediate form of FRS between non-invasive and invasive FRS and suggested a “preinvasive” subtype of fungal sinusitis. The cases in that study presented clinically as invasive FRS; however, histopathologic findings did not show direct angioinvasion and demonstrated wide extension beyond the submucosa. All cases were successfully treated with limited surgical procedures and short antifungal therapy courses. The authors suggested that treatment for these patients falls into an undefined zone between the treatment for fungal balls and that for aggressive FRS [17]. Although the present study also similarly evaluated patients with a condition that fell between fungal balls and invasive FRS, the clinical and pathologic characteristics of our patients differed from those of Paknezhad et al.’s study [17]. 

The limitation of the present study was its retrospective nature and the small number of patients over a long period. Another limitation is that we could not characterize microinvasive FRS although we identified a specific patient group as such. FRS with mucosal invasion in this study may have been found in the process of detecting invasive FRS, but some may resolve spontaneously regardless of whether further treatment is administered. Therefore, further multicenter prospective studies with repeat biopsy are needed to understand the clinical characteristics of microinvasive FRS.

In conclusion, we presented one subtype of FRS that had not been classified under the current guidelines. We identified 13 patients with fungal balls with mucosal invasion but without angioinvasion. Because this presentation represents a boundary between fungal balls and invasive FRS with a likely progression to invasive FRS in future, the definition of microinvasive FRS may be considered. Furthermore, adjuvant antifungal treatment may be considered in immunocompromised hosts with microinvasive FRS.

## Figures and Tables

**Table 1:** Baseline Characteristics and Clinical Courses of the Microinvasive Fungal Rhinosinusitis.

Patient Number	Age (Years)	Sex	Location	Species	Underlying Disease	Symptoms	Duration of Symptoms (Months)	Antifungal Therapy	Disease-Free State at Last Visit	Duration of Follow-Up (Months)
1	57	F	(L) MS	*Aspergillus*		Facial pain	1.5	ND	Yes	20
2	82	F	(R) MS	*Aspergillus*	DM	Nasal obstruction	2	Itraconazole 8 weeks	Yes	21
3	83	M	(L) SS	*Aspergillus*	DM	None		Voriconazole 17 weeks	Yes	22
4	63	M	(L) MS	*Aspergillus*		Postnasal drip	4	ND	Yes	22
5	67	F	(L) MS, ES	*Aspergillus*	DM	Headache	120	Amphotericin B 3 weeks, voriconazole 7 weeks & caspofungin 3 weeks	Yes	23
6	48	M	(R) SS	*Aspergillus*	Acute biphasic leukemia	None		Amphotericin B 8 weeks	Yes	24
7	64	F	(R) MS, ES	*Aspergillus*		Purulent rhinorrhea	1	ND	Yes	22
8	72	F	(R) MS, ES	*Aspergillus*		Postnasal drip	12	ND	Yes	25
9	62	F	(L) MS	*Aspergillus*		Purulent rhinorrhea	27	ND	Yes	43
10	68	M	(R) MS	*Aspergillus*		None		ND	Yes	49
11	61	F	(R) MS	*Aspergillus*		None		ND	Yes	117
12	77	F	(L) MS	*Aspergillus*	Diffuse large B cell lymphoma	None		Amphotericin B 2 weeks & voriconazole 15 weeks	Yes	122
13	71	F	(B) SS	*Aspergillus*		Purulent rhinorrhea	30	ND	Yes	30

Abbreviations: DM, diabetes mellitus; ES, ethmoid sinus; F, female; L, left; M, male; MS, maxillary sinus; ND; not done; R, right; SS, sphenoid sinus.
